# *POLG* ‐Related Parkinsonism with Good Response to Deep Brain Stimulation

**DOI:** 10.1002/mdc3.70588

**Published:** 2026-03-18

**Authors:** Evdokia Efthymiou, Fabian Büchele, Sujitha Mahendran, Lennart Stieglitz, Bettina Balint

**Affiliations:** ^1^ Department of Neurology University Hospital Zurich Zurich Switzerland; ^2^ Department of Neurosurgery University Hospital Zurich Zurich Switzerland; ^3^ University of Zurich Zurich Switzerland

**Keywords:** deep brain stimulation, subthalamic nucleus, young‐onset Parkinson's disease, POLG mutation, mitochondrial disorder

Deep brain stimulation (DBS) is an established treatment for Parkinson's disease (PD), both sporadic and genetic forms^1^, ^2^. Its usefulness in rare genetic parkinsonian conditions is less clear. Here, we describe our experience with polymerase gamma (*POLG*)‐related parkinsonism treated with DBS of the subthalamic nucleus (STN).

## Case Report

A 42‐year‐old woman, diagnosed with young‐onset PD and with negative family history for neurological diseases, was referred for evaluation of DBS. At age 30, she had developed akinetic‐rigid parkinsonism with a good response to levodopa. Later on, motor fluctuations emerged, with levodopa‐induced dyskinesias and marked OFF freezing of gait (FOG) with 2 h OFF period per day. Upon clinical evaluation 12 years after symptom onset, she had asymmetric, akinetic‐rigid parkinsonism (Movement Disorder Society–Unified Parkinson's Disease Rating Scale [MDS‐UPDRS] motor score, part III: 29 points). She was of short stature and additionally exhibited bilateral ptosis (for which she had previously received corrective left eyelid surgery), external ophthalmoplegia, blepharospasm, proximal muscle weakness, reduced deep tendon reflexes and gait ataxia (Video 1). These findings raised suspicion of a mitochondrial disorder, further strengthened by a history of bilateral cataract surgery. Genetic testing revealed a rare heterozygous pathogenic variant in mitochondrial polymerase gamma (*POLG; c.2828G) > A (p.Arg943His*)^3^.

**Video 1:** Clinical features characteristic of POLG‐related disorder. The patient exhibits bilateral ptosis and external ophthalmoplegia, accompanied by proximal muscle weakness affecting the neck, upper, and lower limbs. Additional features include short stature and gait ataxia. Dystonia is present in form of blepharospasm.

Dopamine Transporter Scan (DaTSCAN) imaging showed a symmetrical decrease in tracer uptake. Brain MRI showed mild general atrophy. Other investigations, including non‐ischemic forearm exercise test (NIFET), sub‐anaerobic threshold exercise test (SATET), electroencephalogram (EEG), cerebrospinal fluid, polysomnography and electroneuromyography, were non‐contributory. Neuropsychological testing revealed predominantly fronto‐subcortical and possibly mesiotemporal, dysfunction characterized by verbosity, disinhibition, and deficits in attentional, mnestic, and executive subdomains. A levodopa challenge with 250 mg levodopa demonstrated good dopa‐responsiveness (MDS‐UPDRS part III: OFF 54 points; ON 23 points; Video 2a: OFF, 2b: ON), with troublesome dyskinesias. Based on the PD‐like characteristics of her parkinsonism, given the normal MoCA score (28 points), the informant report indicating no cognitive difficulties in daily life, and the patient's clear wish to reduce medication a joint decision for DBS in the STN was made. Directional brain electrodes were implanted stereotactically in each STN (Medtronic B35200, Percept PC, Sensight electrodes B33005). Six months post‐DBS, there were no longer OFF periods or FOG, and reduction of levodopa led to a nearly complete cessation of dyskinesias (Video 2c). To date, 48 months post‐DBS, occasional dyskinesias are present but not perceived by the patient, who takes levodopa only in the evening (Video 2d). The only complaint 6 months after the surgery was mild gait ataxia, which persists at 48 months, along with occasional—partially levodopa responsive—freezing of gait in crowded environments. Although neuropsychological testing revealed a marked decline in attention, memory, and executive functions even before the surgery, with further deterioration across all functional domains postoperatively, the patient has not reported, and does not currently experience, any limitations in daily life, nor have any been noted by relatives. Further details on the clinical course and stimulation parameters are provided in Table 1.

**Video 2:** Preoperative and postoperative assessment. (a) After 12 h of dopaminergic medication withdrawal, the patient is markedly parkinsonian with significant bradykinesia. Independent gait is not possible. (b) Assessment of MDS‐UPDRS part III following administration of 250 mg levodopa. The patient shows generalized, troublesome dyskinesias along with a marked improvement in bradykinesia. Standing up and walking are possible without assistance. (c) Six months after DBS: the patient shows sustained improvement in bradykinesia and gait, with only mild dyskinesias primarily affecting the orofacial region. (d) Forty‐eight months after DBS: the patient continues to demonstrate stable motor function with mild gait ataxia including postural instability when turning.

**TABLE 1:** Course of drug treatment, MDS‐UPDRS, stimulation parameters and MoCA

	Pre‐DBS		6 months post‐DBS	48 months post‐DBS
LEDD (mg)	555		100	75
MDS‐UPDRS I	9		8	3
MDS‐UPDRS II	9		3	2
MDS‐UPDRSIII (OFF: 12 h washout)	L‐Dopa OFF: 54	L‐Dopa ON: 29	L‐Dopa ON/DBS‐ON: 24	L‐Dopa ON/DBS‐ON: 22
MDS‐UPDRS IV	7		0	1
Stimulation parameters			STN left: contact 2‐ C+, 2 mA, 60 μs, 130 Hz STN right: contact 10‐ C+, 2 mA, 60 μs, 130 Hz	STN left: contact 3‐ C+, 3,75 mA, 60 μs, 130 Hz STN right: contact 10b‐ C+, 1,1 mA, 60 μs, 130 Hz
MoCA‐Test	28		20	19
Neuropsychological testing	Moderate neuropsychological impairment: predominantly fronto‐subcortical and possibly mesiotemporal, dysfunction		Moderate neuropsychological impairment: clear deterioration in attention, memory, and executive function compared to the pre‐DBS assessment	Moderate to marked neuropsychological impairment: further deterioration across all cognitive domains, compared to 6 months post‐DBS assessment. Additionally, enhancement of the behavioral dystegulation (reduced social distance and increased impulsivity)

*Note*: SVNP (“Schweizerische Vereinigung der Neuropsychologen und Neuropsychologinnen”) criteria were applied for the severity level of the neuropsychological impairment^10^.

Abbreviations: DBS, deep brain stimulation; LEED, Levodopa equivalent daily dose; MDS‐UPDRS, Movement Disorders Society United Parkinson Disease Rating Scale; MoCA‐Test, Montreal Cognitive Assessment Test; STN, subthalamic nucleus.

## Discussion

Parkinsonism in *POLG*‐related disease, associated with either dominant or recessive pathogenic variants has been previously reported, either with clear clues (Table 2, red flags for *POLG*‐related parkinsonism) to mitochondrial disease^4^, ^5^; or with a relatively pure phenotype^6^. Neuropathology shows loss of pigmented neurons and pigment phagocytosis in substantia nigra without Lewy bodies^7^. *POLG*‐related parkinsonism is typically dopa‐responsive; however, we present here the first report of STN‐DBS for managing motor fluctuations in this entity.

**TABLE 2:** Features of *POLG*‐related parkinsonism

Clues that might point to and red flags for *POLG*‐related parkinsonism:
Early onset (~40 years or earlier) Premature ovarian failure, hypogonadism progressive external ophthalmoplegia, ptosis myopathy, peripheral neuropathy, cerebellar ataxia, epilepsy, seizures, stroke‐like episodes, migraine retinopathy, sensorineural hearing loss Cataracts Short stature Psychiatric comorbidities (in particular, depression) Liver dysfunction Renal dysfunction Diabetes mellitus Gastrointestinal dysmotility Family history with one or more features of the above, or inherited “multiple sclerosis‐like illness”
Implications for management:
Parkinsonism is typically responsive to dopaminergic therapy Valproic acid and sodium divalproate should be avoided (risk of liver failure)

Abbreviation: *POLG*, polymerase gamma.

Overall, the outcome was favorable with a significant reduction in motor fluctuations and improved quality of life, alongside a reduced requirement of dopaminergic medication and the recovery of independence in daily life. It has been suggested that chronic administration of levodopa can cause alterations in mitochondrial respiratory chain activity, likely related to oxidative stress provoked by increased dopamine turnover^8^. The same mechanism may exaggerate a mitochondrial defect already present. Hence, saving levodopa with STN‐DBS might have particularly beneficial effects in *POLG*‐related parkinsonism. However, there was a cognitive decline during follow up, although not impacting on day‐to‐day life. It remains unclear to what extent subthalamic stimulation contributed to the cognitive decline, or whether natural disease progression played the predominant role^9^. To explore this further is relevant, as a different DBS target (globus pallidus internus) or other therapeutic approaches such as dopaminergic pump therapy might then be a preferable option in such cases. Further follow‐up and cases will allow us to deepen our understanding of DBS targeting and outcomes in patients with genetic Parkinsonian syndromes like *POLG* mutations.

## Author Roles

(1) Research project: A. Conception, B. Organization, C. Execution; (2) Statistical Analysis: A. Design, B. Execution, C. Review and Critique; (3) Manuscript Preparation: A. Writing of the first draft, B. Review and Critique.

EE: 1A, 1B, 1C, 3A.

BB: 1A, 1B, 3A, 3B.

FB: 3B.

SM: 3B.

LS: 3B.

## Disclosures

**Ethical Compliance Statement:** We hereby confirm that the present study conforms to the ethical standards and guidelines of the Journal. The authors confirm that the approval of an institutional review board was not required for this work. The patient has given written and informed consent for online publication of their videos.

**Funding Sources and Conflict of Interest:** No specific funding was received for this work. The authors declare that there are no conflicts of interest relevant to this work.

**Financial Disclosures for the previous 12 months:** BB has received funding from the Michael J. Fox Foundation, the Hurka Foundation, the Olga Mayenfisch Foundation and the Betty and David Koetser Foundation, and has received royalties from Oxford University Press. EE has received funding from Medtronic and served on the advisory boards for Lecigon (Spirig Pharma). She has received honoraria for teaching activities from the “Forum für medizinische Fortbildung” and for Workshop (Bial Pharma). FB received honoraria for teaching activities for “Forum für medizinische Fortbildung.” SM has received funding from the Betty and David Koetser Foundation and honoraria for teaching activities from “Forum für medizinische Fortbidung.” LS received lecture fees by Medtronic and is member of the Medtronic YNTP faculty. He received honoraria as member of the scientific advisory board from Medtronic, Insightec and Admirabiles.

## Data Availability

The data that support the findings of this study are available on request from the corresponding author. The data are not publicly available due to privacy or ethical restrictions.
